# Generating a Knockdown Transgene against *Drosophila* Heterochromatic *Tim17b* Gene Encoding Mitochondrial Translocase Subunit

**DOI:** 10.1371/journal.pone.0025945

**Published:** 2011-10-06

**Authors:** Mikael Garabedian, Michael Jarnik, Elena Kotova, Alexei V. Tulin

**Affiliations:** Fox Chase Cancer Center, Philadelphia, Pennsylvania, United States of America; University of Crete, Greece

## Abstract

Heterochromatic regions of eukaryotic genomes contain multiple functional elements involved in chromosomal dynamics, as well as multiple housekeeping genes. Cytological and molecular peculiarities of heterochromatic loci complicate genetic studies based on standard approaches developed using euchromatic genes. Here, we report the development of an RNAi-based knockdown transgenic construct and red fluorescent reporter transgene for a small gene, *Tim17b*, which localizes in constitutive heterochromatin of *Drosophila melanogaster* third chromosome and encodes a mitochondrial translocase subunit. We demonstrate that Tim17b protein is required strictly for protein delivery to mitochondrial matrix. Knockdown of *Tim17b* completely disrupts functions of the mitochondrial translocase complex. Using fluorescent recovery after photobleaching assay, we show that Tim17b protein has a very stable localization in the membranes of the mitochondrial network and that its exchange rate is close to zero when compared with soluble proteins of mitochondrial matrix. These results confirm that we have developed comprehensive tools to study functions of heterochromatic *Tim17b* gene.

## Introduction

Despite ongoing intensive research since the nineteenth century, heterochromatin remains a mysterious domain of the genome. In 1933, Emil Heitz described distinct blocks of chromatin in the nuclei of *Drosophila* cells that he termed “heterochromatin” [1]. Over the years, it has become clear that heterochromatin is a ubiquitous component of all eukaryotic genomes [2], and, in some cases, heterochromatic DNA encompasses up to 90% of the genome. Genetic analysis and sequencing of heterochromatin have shown that the major portion of heterochromatin consists of the so-called satellite repeats [3], [4], [5] or tandem arrays that have a specific short sequence ranging in length from two pairs to several hundred pairs of nucleotides. Extensive circuits of such satellites are located in centromeric regions. Moderate repeats are another abundant class of elements that are also associated with heterochromatin. This large subclass consists of mobile genetic elements (ME) [6], [7]. Besides satellite arrays and mobile elements, heterochromatin contains “infrequent” unique genes [8], which can be amplified and assembled into tandem arrays [9]. However, the abundance of such repeated DNA and the “highly diluted” amount of unique sequences complicate standard structural and genetic analysis of heterochromatin [10], making these areas of the genome “terra incognita” for molecular genetics. Furthermore, heterochromatin seems to have earned a reputation as “junk genomic DNA” in the scientific community, thus making the study of heterochromatin unpopular. Notwithstanding these drawbacks, a growing body of evidence indicates that heterochromatin plays a crucial role in a cell's life cycle [11], [12]. Heterochromatin contains functional elements of chromosomes, centromeres, and telomeres [13], [14], [15], as well as ribosomal DNA arrays [16]. Moreover, most of the unique heterochromatic genes appear to be the housekeeping genes [17], [18]. Therefore, the study of heterochromatin, though technically complicated, is important.

**Figure 1:**
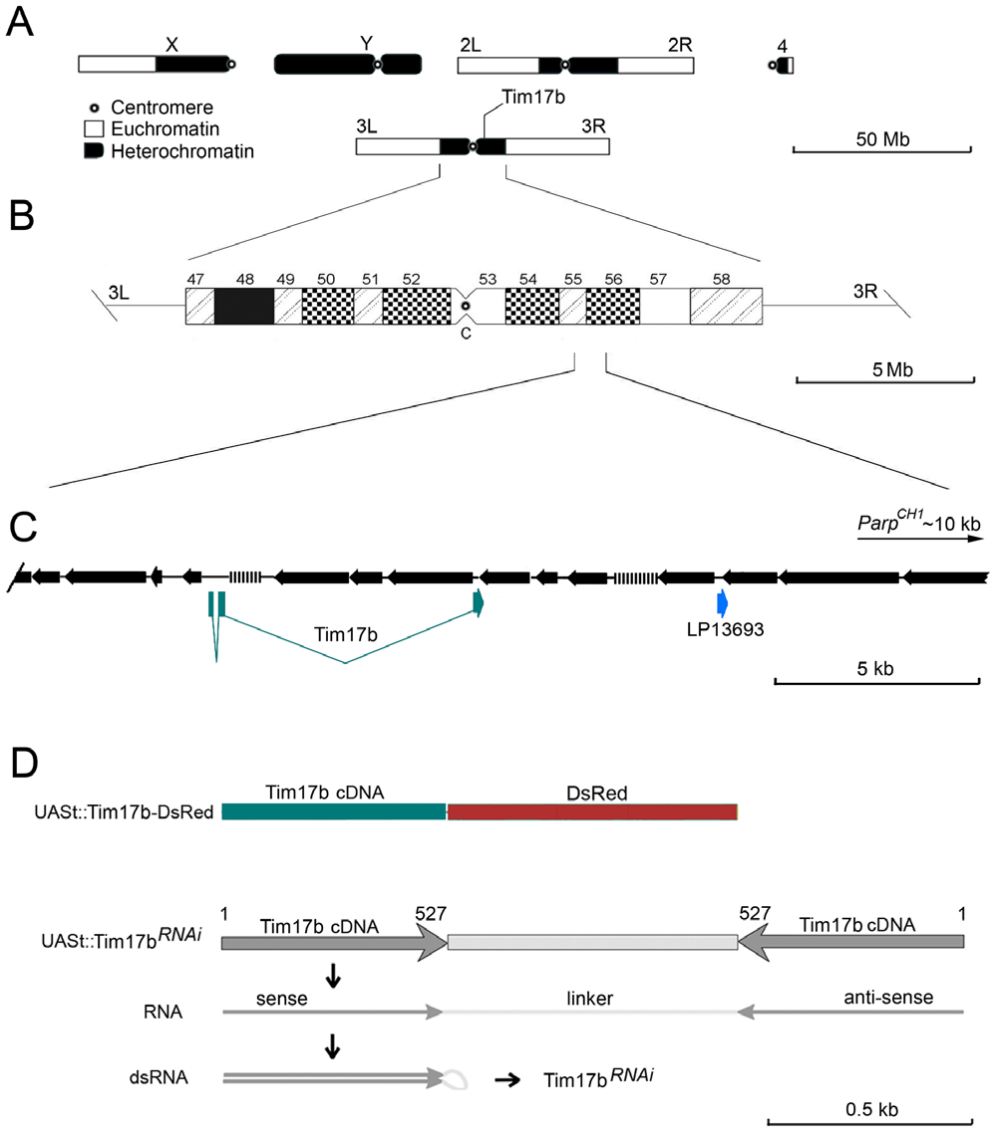
*Tim17b* gene is located within constitutive heterochromatin of third chromosome. **A.** Schematic illustration of *Drosophila melanogaster* chromosomes. Heterochromatic regions are shown in black. **B.** An ideogram of chromosome 3 heterochromatin. **C.** A scheme summarizing the sequence organization around *Tim17b* locus. Retrotransposable and transposable repeated DNA are shown by black arrows (arrowhead – 3′ end). Genes identified by homology to known cDNAs are shown below (blue boxes correspond to exons). Distance to closest characterized locus (*Parp*) is indicated. **D.** Generation of *UAS-Tim17b-DsRed* and *UAS-Tim17b^RNAi^* transgenes. Structure of transgenic constructs is shown. Nucleotide positions of Tim17b cDNA are indicated.

In *Drosophila*, heterochromatin takes up about 30% of the genome [3], [5] (Figure 1A). These genomic areas have suppressed meiotic homology-dependent recombination [19], [20]. Therefore, homology-targeted mutagenesis [21] cannot be applied for heterochromatic genes. The high level of dilution by repeated DNA and transcriptional silencing within heterochromatin also complicate standard random insertional mutagenesis [22], [23]. Therefore, the most obvious method of targeting specific genes within heterochromatin would use an RNAi knockdown strategy [24], [25].

In this study, we applied a snap-back transgenic RNAi approach [24], [25] to target a small heterochromatic gene, *Tim17b*, which is located in constitutive heterochromatin of the third chromosome near the previously characterized *Parp1* locus (Figure 1B and C). *Tim17b* is a typical heterochromatic locus located in a previously well-characterized region [18], where unique exonic sequences are separated by tandem arrays of repeated DNA (Figure 1C). *Tim17b* is a relatively small locus which spans less than 5.5 kb of genomic DNA, whereas exons take up only 0.5 kb, and promoter elements characteristic of euchromatic genes are not detectable. Thus, it is unrealistic to suggest that random insertional mutagenesis projects [22], [23] will ever target it. *Tim17b* encodes a mitochondrial translocase subunit, which is involved in the delivery of proteins to the mitochondrial matrix [26]. The Tim17b protein superfamily demonstrates high evolutionary conservation in eukaryotes (Figure S1A), suggesting that the functions performed by Tim17b in mitochondria are also conserved and very important. In addition to the *Tim17b* gene in heterochromatin of third chromosome, the *Drosophila* genome contains two more *Tim17b*-like genes (Figure S1B), CG1158 and CG15257, encoding Tim17b1 and Tim17b2 proteins and correspondingly localized in euchromatin of 3R and 2L chromosomal arms. However, we found that only Tim17b expresses ubiquitously (Figure S1C), while the other two are produced only in adult male tissues. Since Tim17b most likely plays a major functional role in the translocase complex, we have focused our research on this protein. Studies of mitochondria, in general, and mitochondrial translocase complexes, in particular, are very important because they allow us to understand the roles of mitochondria in longevity, apoptosis, cellular senescence, and tumorigenesis [27]–[30], as well as permit the development of new therapeutic drugs. Therefore, the reagents and techniques generated and characterized in this study will be useful for analyzing mitochondrial function and regulation, as well as for studying mechanisms of apoptosis.

## Results

### Tim17b-DsRed recombinant protein is localized to mitochondria

**Figure 2:**
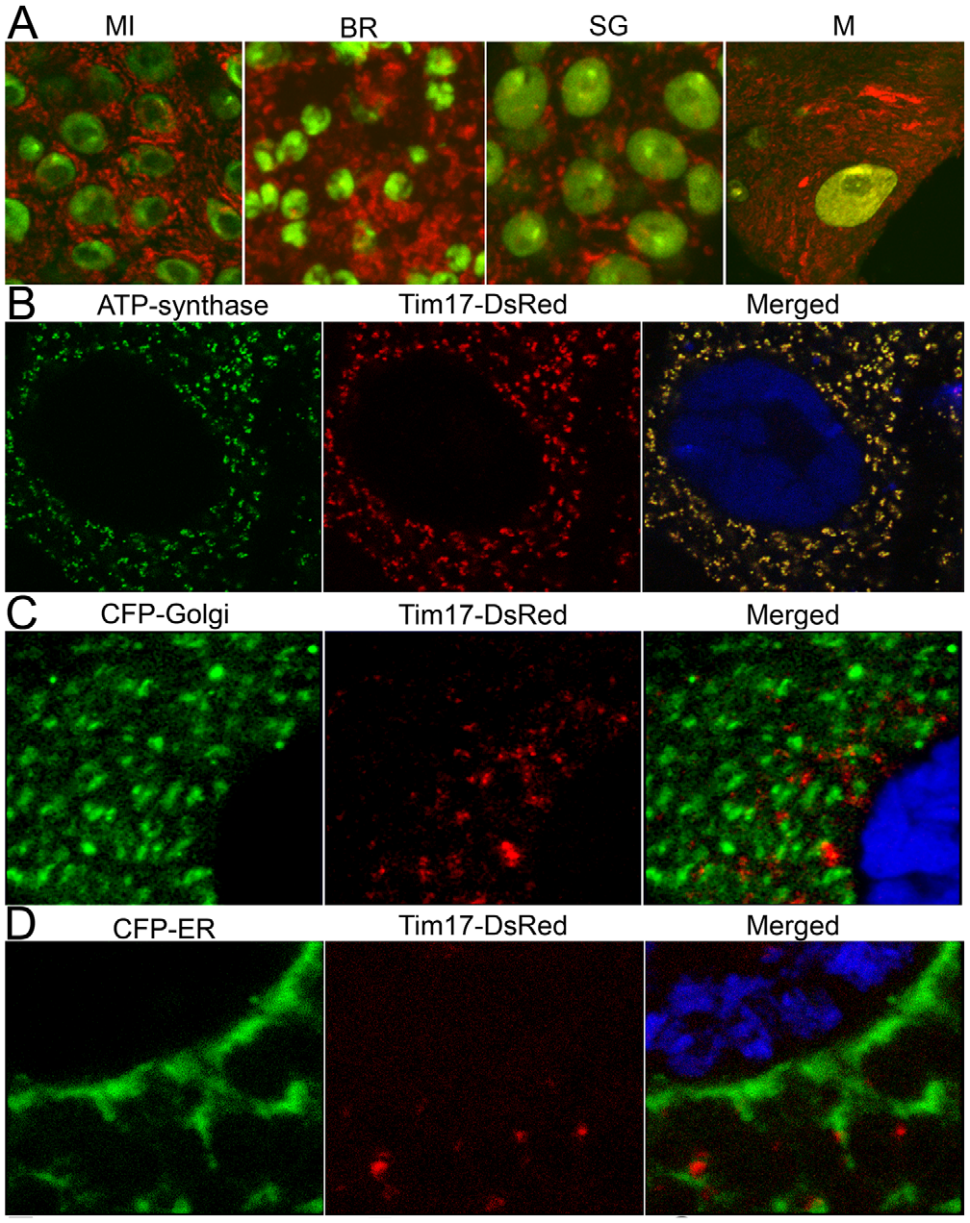
Tim17b-DsRed recombinant protein is localized to mitochondria. **A.** Tim17b-DsRed (red) recombinant protein labels mitochondria in all *Drosophila* tissues. MI – mid-intestine; BR – brain; SG – salivary glands; M – body wall muscles. DNA is stained with OliGreen dye (green). **B–D.** Tim17b-DsRed recombinant protein is co-localized with mitochondrial protein ATP-synthase (**B**), but not with Golgi (**C**) or Endoplasmic Reticulum (ER) (**D**) markers. The dissected larval salivary glands expressing Tim17b-DsRed (red) were stained with anti-ATP-synthase antibody (green) and DNA binding dye Draq5 (blue) (**B**). The dissected larval salivary glands co-expressing Tim17b-DsRed (red) and CFP-Golgi (green) (**C**) or CFP-ER (green) proteins (**D**) were stained with DNA binding dye Draq5 (blue).

Previously, we designed transgenic *Drosophila* expressing a Tim17b-DsRed fluorescent reporter protein [18] (Figure 1D). To analyze further Tim17b expression and protein localization, we expressed a *UAS::Tim17b-DsRed* transgene using ubiquitous GAL4 drivers (described in Materials and Methods). We found that Tim17b-DsRed was enriched in cytoplasmic network-like structures in all *Drosophila* tissues tested in our experiments (Figure 2A). The distribution of Tim17b-DsRed in the cytoplasm precisely matches the localization of mitochondrial protein ATP-synthase (Figure 2B), but it does not overlap with Golgi or endoplasmic reticulum markers (Figure 2C and D). Flies expressing Tim17b-DsRed are viable and do not exhibit any defects in development or fertility. These results allow us to conclude that our transgenic reporter Tim17b-DsRed has proper localization in mitochondria and causes no disruptive effects on mitochondrial functions. Therefore, the Tim17b-DsRed recombinant protein should function properly as an *in vivo* marker of mitochondrial translocase complex.

### Co-expression of *Tim17b-DsRed* and *Tim17b^RNAi^* transgenes diminishes the amount of Tim17b-DsRed protein and disrupts mitochondria

**Figure 3:**
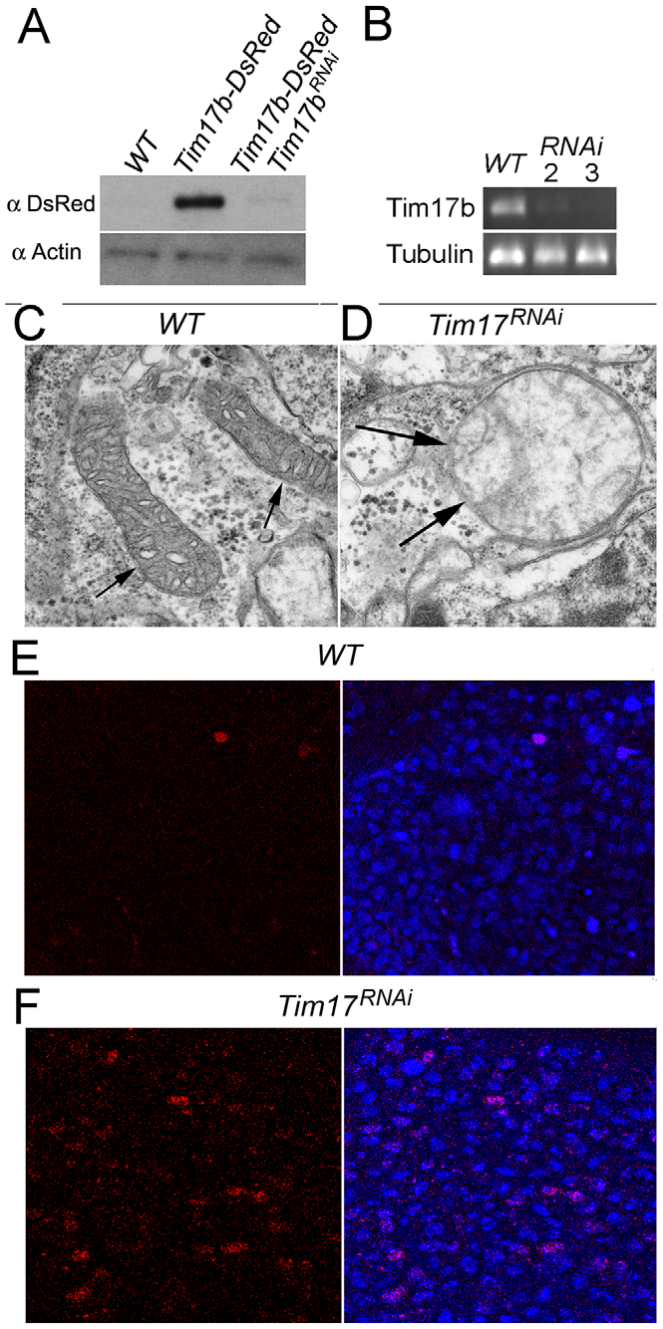
Expression of *Tim17b^RNAi^* transgene disrupts Tim17b protein production. **A.** Co-expression of Tim17b-DsRed recombinant protein with *Tim17b^RNAi^* eliminates Tim17b-DsRed protein production. Western blot hybridization was used to compare the amount of Tim17b-DsRed protein in *Gal4 69B; UAS-Tim17b-DsRed* and *Gal4 69B; UAS-Tim17b-DsRed; UAS-Tim17b^RNAi^* larvae. Anti-Actin antibody was used as a loading control. **B.** Expression of *Tim17b^RNAi^* disrupts intrinsic Tim17b mRNA production. RT-PCR using intrinsic Tim17b-specific primers demonstrates that accumulation of Tim17b mRNA is abolished in *Tim17b^RNAi^*-expressing animals. The numbers 2 and 3 indicate age of animals collected for analysis after egg laying. Primers specific to Tubulin mRNA were used as a loading control. **C**–**D.** Expression of *Tim17b^RNAi^* disrupts mitochondria. The structure of wild-type mitochondria detected by TEM (**C**) is affected in *Tim17b^RNAi^*-expressing animals (**D**). Arrows indicate mitochondria. **E**–**F.** Expression of *Tim17b^RNAi^* increases apoptosis in larval brain. WT – wild- type first-instar larvae (**E**). *Tim17b^RNAi^*-expressing first-instar larvae (**F**). Dissected larval brains were stained using ApopTag (red), which detects apoptotic cells. DNA visualized using OliGreen dye (blue).

In order to disrupt *Tim17b* function, we employed a transgenic RNAi strategy described in [31]. We cloned full-length 0.5 kb Tim17b cDNA in direct and reverse orientation separated by 0.9 kb spacer DNA into pUASt vector (Figure 1D) and used this construct to transform *Drosophila*. The amount of Tim17b-DsRed protein is significantly diminished in animals co-expressing the resultant *Tim17b^RNAi^* transgene with *Tim17b-DsRed* when compared with animals of the same developmental stage expressing only *Tim17b-DsRed* (Figure 3A). Moreover, expression of intrinsic Tim17b is also abolished by Tim17b^RNAi^ (Figure 3B). Most of the animals expressing *Tim17b^RNAi^* were arrested early in the embryonic stage and died; less than 5% of the remaining embryos survived up to later second-instar or third-instar. These results confirm the effectiveness of the *Tim17b^RNAi^* transgene. Therefore, we tested further the effects of *Tim17b^RNAi^* on mitochondria. We dissected mid-intestine from *Tim17b^RNAi^*–expressing surviving second-instar larvae prior to lethal stage and examined these tissues by transmission electron microscopy (TEM) analysis. Strikingly, typical mitochondria (Figure 3C) were scarce in *Tim17b^RNAi^*-expressing cells. Instead, we often observed an abnormal structure surrounded by double membrane with residual cristae inside (Figure 3D). Moreover, we found that expression of *Tim17b^RNAi^* stimulates apoptosis (Figure 3E and F). These results suggest that our *Tim17b^RNAi^* transgene is effective in disrupting mitochondrial function, thereby demonstrating, for the first time, the essential role of the Tim17b protein in mitochondria.

### Expression of *Tim17b^RNAi^* transgene abolishes protein delivery to mitochondrial matrix

**Figure 4:**
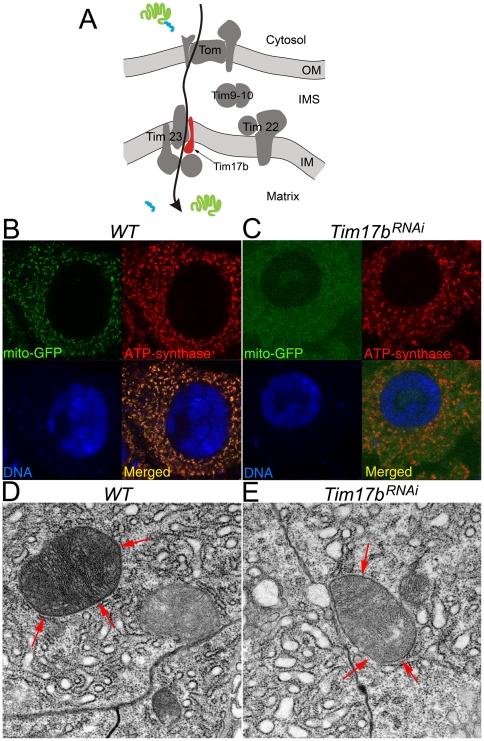
Elimination of Tim17b function by RNAi expression disrupts protein delivery to mitochondrial matrix. **A.** Model of organization and function of mitochondrial translocase complexes TOM, TIM9-10, TIM23 and TIM22. Arrow shows pathway of protein with cleavable N-terminal signal translocation through TOM-TIM23 into matrix. Position of Tim17b protein (red) in TIM23 complex is shown. OM – outer membrane; IMS – intermembrane space; IM – inner membrane. Green – protein of mitochondrial matrix. Blue – cleavable N-terminal signal peptide. **B–C.** Tim17 is required for *mito-GFP* delivery to mitochondria. The dissected larval mid-intestine expressing *mito-GFP* (green) (**B**) or co-expressing *mito-GFP* (green) with *Tim17b^RNAi^* (**C**) were stained with anti-ATP-synthase antibody (red) and TOTO3 DNA binding dye (blue). Displacement of *mito-GFP* from mitochondria is clearly seen in *Tim17b^RNAi^*–expressing tissues. **D** *–* **E.** TEM analysis of tissues shown in **B** *–* **C** panels demonstrates that mitochondrial structure has still not been compromised by the time *mito-GPF* delivery to mitochondria is disrupted. Red arrows indicate mitochondria.

According to previous studies, the Tim17b protein belongs to the inner membrane translocase complex Tim23, which is involved in the delivery of protein with specific cleavable N-terminal peptide signal to the mitochondrial matrix and inner membrane [26] (Figure 4A). Therefore, we tested whether the expression of *Tim17b^RNAi^* transgene would disrupt this pathway. To track protein delivery into mitochondrial matrix, we used a transgenic *Drosophila* expressing *UAS::mito-GFP* reporter [32]. This recombinant protein carries the N-terminal signal of delivery into mitochondria [32]; upon expression in wild-type *Drosophila*, it accumulates in mitochondria and co-localizes with mitochondrial protein ATP-synthase (Figure 4B). However, using inducible heat shock hs::GAL4 driver (see Materials and Methods for details), co-expression of *Tim17b^RNAi^* and *mito-GFP* in third-instar larvae resulted in the accumulation of *mito-GFP* in the cytosol, but not colocalization with ATP-synthase (Figure 4C). ATP-synthase is a stable intrinsic transmembrane mitochondrial protein expressed early during the larval development. Therefore, when hs::GAL4 driver induces co-expression of both *Tim17b^RNAi^* and *mito-GFP* in third-instar larvae, ATP-synthase is already localized properly inside the mitochondrial membrane. While disruption of Tim17b protein production by *Tim17b^RNAi^* expression precludes mito-GFP delivery into mitochondria, the localization of ATP-synthase, which is expressed earlier during the larval development, is not affected. Therefore, even though expression of *Tim17b^RNAi^* in third-instar larvae blocks delivery of new mitochondrial proteins (*mito-GFP*), proper localization of ATP-synthase demonstrates that the mitochondrial structure itself was not affected (Figure 4D and E). This last finding suggests that expression of *Tim17b^RNAi^* can allow analyzing Tim23 translocase in a spatially and temporally controlled manner, without broad cytotoxic effects.

### Tim17b-DsRed fluorescent protein represents a useful tool for the study of *in vivo* mitochondrial dynamics

The results described above indicate that the fluorescent reporter protein Tim17b-DsRed and *Tim17b^RNAi^* transgene are sufficient for both the detection of translocase protein localization and functional analysis of the translocase complex. Further, we tested the capability of Tim17b-DsRed reporter for *in vivo* assays.

**Figure 5:**
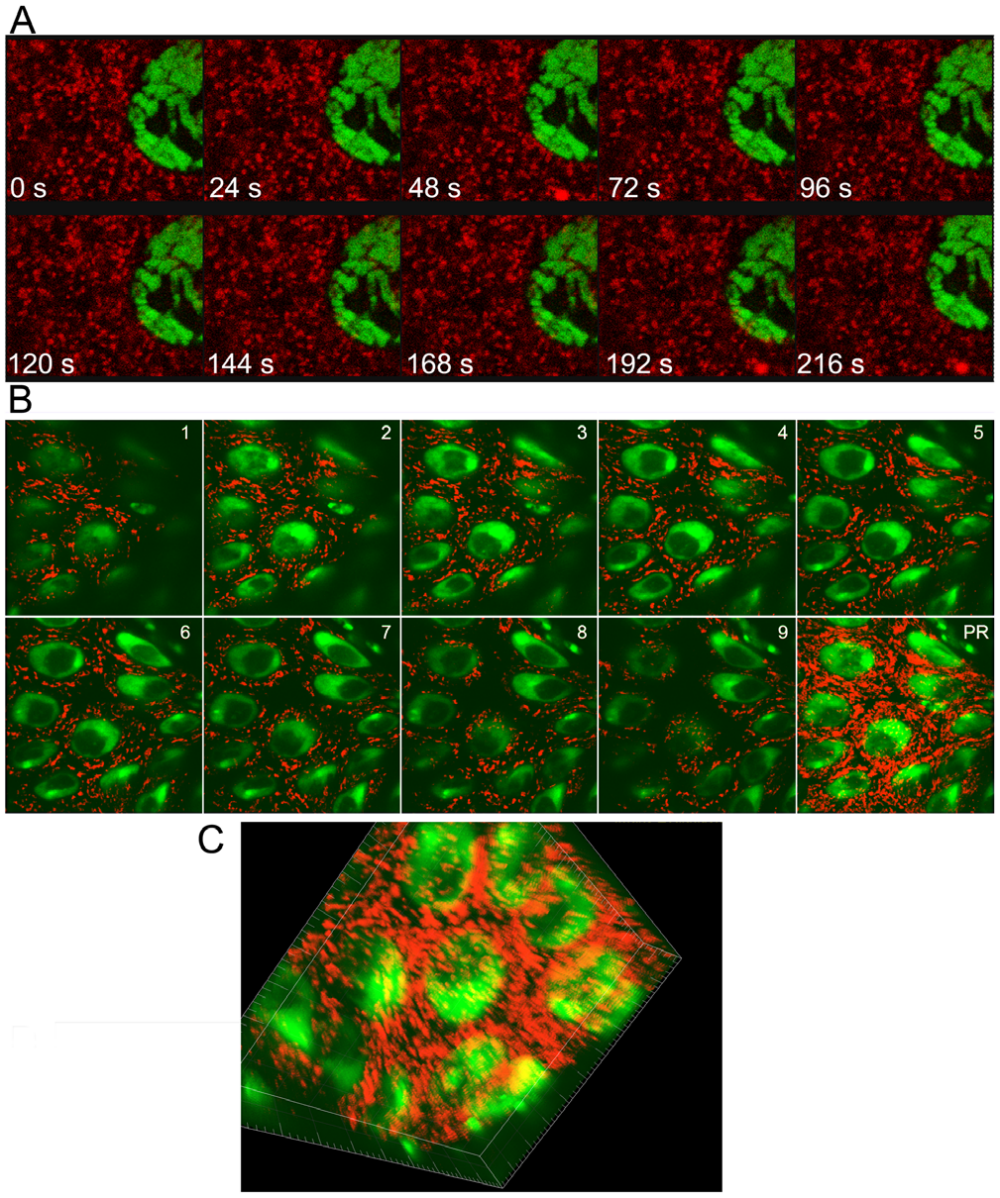
Use of Tim17b-DsRed fluorescent reporter for time-lapse microscopy of mitochondria, 3D reconstruction of mitochondrial network and mitochondrial proteins dynamics assay. **A.** Time-lapse microscopy of live *Drosophila* tissue. Tim17b-DsRed is shown in red color. DNA is detected using Draq5 dye (green). **B–C.** 3D-mitochondrial network within mid-intestinal cells. 1–9 represent individual confocal sections. PR – X-Y projection. **C.** 3D reconstruction of the mitochondrial network is presented. Tim17b-DsRed is shown in red color. DNA is detected using TOTO3 dye (green).

**Figure 6:**
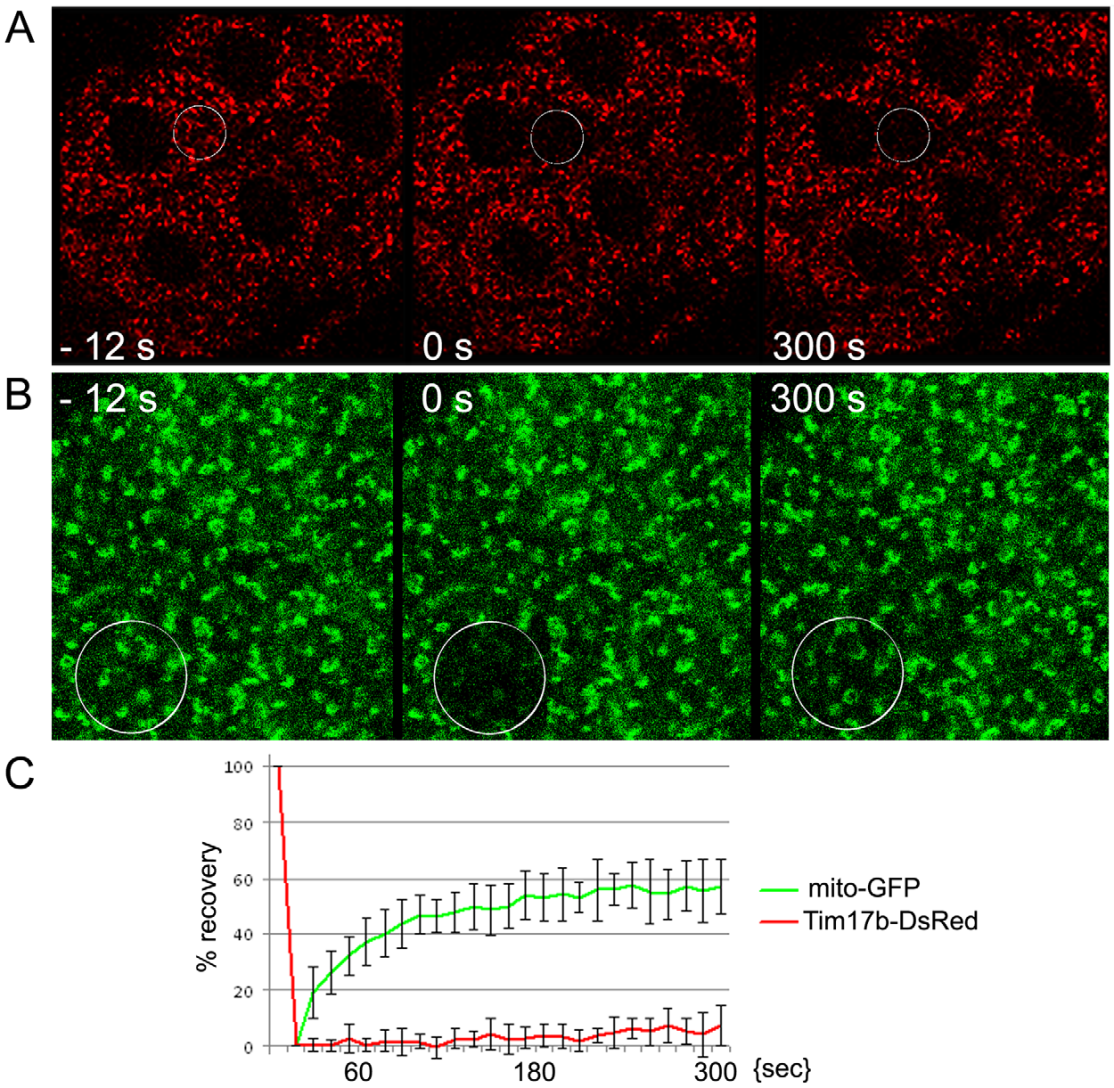
Fluorescence Recovery After Photobleaching (FRAP) assay for Tim17b-DsRed and mito-GFP proteins. **A.** Tim17b-DsRed (red). **B.** *mito-GFP* (green). White circle shows position of photobleached area (**A** *–* **B**). In contrast to soluble GFP protein of mitochondrial matrix, assay demonstrates that Tim17b, which is a transmembrane protein, has a very slow replacement/dynamic rate (**C**).

In developing *Drosophila,* mitochondria have been studied using immunohistochemical approaches [33], as well as GFP-tagged reporters [32]. Here, we developed a method to visualize live mitochondria using a red fluorescent reporter and high resolution confocal microscopy. By visualizing mitochondria within living tissue dissected from strains expressing Tim17b-DsRed, we can study dynamic mitochondrial behavior. Time-lapse confocal microscopy, as shown in Figure 5A, demonstrates the ability to track the migration of a single mitochondria in the cytoplasm of larval salivary glands. In many metabolically active tissues, mitochondria form a network where individual mitochondria are interconnected [34]. In order to find these mega-organelles, we examined live larval tissues expressing the Tim17b-DsRed reporter. We found that cells of the larval intestine contain such interconnected mitochondria (Figure 5B and C). The interconnection of organelles to a single network suggests the ability of individual mitochondria to exchange soluble proteins of nucleoplasm. To test this hypothesis, we employed the Fluorescence Recovery After Photobleaching Assay (FRAP). We compared recovery rates for a soluble protein of the mitochondrial matrix (*mito-GFP*) and the transmembrane protein Tim17b-DsRed (Figure 6). Although *mito-GFP* rapidly recovered after photobleaching in part of the mitochondrial network (Figure 6B), Tim17b-DsRed protein showed almost no recovery (Figure 6A). These results indicate that transmembrane proteins of translocase complexes are stably positioned in the individual mitochondrial unit and that the exchange rate for these proteins is very low.

## Discussion

Heterochromatic regions comprise a considerable portion of eukaryotic genomes. This compartment of the genome contains a number of genetic loci that are just as important as euchromatic genes [12], [35], [36], yet they are inaccessible for study by more conventional methods. One of the hallmark features of heterochromatic DNA is its strong enrichment in repetitive sequences. This property of heterochromatin complicates further the functional and structural analysis of heterochromatic loci. In *Drosophila melanogaster*, approximately 30% of genomic sequences are heterochromatic. Heterochromatin includes the entire Y-chromosome and 50% of the X-chromosome, as well as 25% of peri-centromeric chromatin of autosomes 2 and 3 (Figure 1A) [10]. With complete sequencing of the *Drosophila* genome, it has become extremely important to develop and test strategies for targeting specific genes located in heterochromatin. In this study, we described the molecular genetics tools which target a small protein encoding gene, *Tim17b*, located in centromeric heterochromatin of the third chromosome.

Taken together, our findings demonstrate that an RNAi knockdown strategy based on designing a snap-back transgene can be successfully applied for targeting heterochromatic genes. By ubiquitous expression of *Tim17b^RNAi^* transgene, we were able to disrupt mitochondria in an organism-wide manner (Figure 3), using inducible driver functions of mitochondrial translocase which could be affected at specific age-dependent developmental stages (Figure 4). Collection of tissue-specific GAL4 drivers affords the capability to express the *UAS::Tim17b^RNAi^* transgene in specific tissues or even in specific cells, thereby allowing the study of mitochondrial function. Depletion of Tim17 protein by *Tim17b^RNAi^* transgene expression leads to lethality and apoptotic-like phenotypes (Figure 3). Therefore, the combination of *Tim17b^RNAi^* transgene with tissue-specific GAL4 drivers offers tools for eliminating targeted cells. This technique may be useful in a wide range of studies targeting development and organogenesis, as well as mitochondrial biology.

Mitochondria contain highly complex translocase machinery involved in the transport of proteins through the double membrane bilayer. Metabolic and genetic disorders in mitochondrial structure and/or function have been linked to a number of human diseases including cancer [27]–[29], [37]. In fact, out of 4 million children born each year in the United States, 4000 develop diseases which can be correlated with mitochondrial dysfunction [27]. Cancerous cells, in particular, have been shown to have a range of distinct defects in mitochondrial structure and metabolism. Various cancer cell lines show major differences in terms of the number, size, shape, and overall metabolism of mitochondria [38], [39], [40]. Mitochondria found in rapidly growing tumors show a tendency to present fewer and smaller mitochondria with fewer cristae, suggesting a disruption in structure at the molecular level. In fact, changes to the molecular structure of the mitochondrial inner membrane have been found in cancer cell lines, including a shortfall of ATPase components.

Although disruption of mitochondrial structure and metabolism has been linked to cancer, no universal metabolic alteration characteristic of all tumor lines has been identified in mitochondria [27]. As demonstrated by TEM data, our RNAi knockout of the Tim17b subunit of the Tim23 complex shows significant disruption of inner membrane structure and cristae formation of mitochondria. In addition, this disruption completely eliminates protein transport into the mitochondrial network. When combined with the red fluorescent reporter transgene for Tim17b, this technique becomes a highly useful tool for studying the Tim23 translocase complex directly. We propose that this tool can be also applied for analysis of mitochondrial metabolism in cancer cells, eventually leading to the development of more targeted therapeutic treatments of cancer.

## Materials and Methods

### *Drosophila* strains and genetics

Flies were cultured on standard cornmeal-molasses-agar media at 22–25°C. The fly stocks were generated by the standard genetic methods or obtained from the Bloomington *Drosophila* Stock Center, except as indicated. Genetic markers are described in Flybase [41]. pP{w1, UAS::Tim17-DsRed}, called UAS:: Tim17-DsRed, was described in [18]. pP{w1, UAS::mito-GFP}, called mito-GFP, was described in [32]. The following GAL4 driver strains were used: arm::GAL4 (Bloomington stock #1560), 69B-GAL4 [42], and hs::Gal4, which was a gift from the G. Cavalli lab. To induce expression from the hs::Gal4 driver, *Drosophila* larvae were heat-shocked for 1 hr at 37°C twice daily for three days prior to the late third-instar stage; then tissues were dissected for protein localization and dynamics analysis.

### Construction of transgenic *Drosophila*

To construct the anti-Tim17b RNAi transgene, we cloned a 693-bp fragment of Tim17b cDNA (from CK01513 clone) in direct and inverted orientation within the pUASt vector. As a spacer between inverted repeats, we used a 720-bp fragment of DsRed sequence (Clontech) (Figure 1D). Transformation was as described [43], with modifications [44].

### Western blot

The following antibodies were used for immunoblotting assays: anti-DsRed (rabbit, 1:1000, Clontech # 8370-1) and anti-Actin (mouse, 1:1000, Chemicon). Western blotting was done using the detection kit from Amersham/GE Healthcare (#RPN2106), according to manufacturer's instructions.

**Electron microscopy** (as described in [45]).

### Immunostaining of *Drosophila* larval tissues

Larval tissues were dissected in Grace's medium brought to room temperature. Samples were then moved directly into fixative solution of 4% formaldehyde in PBS containing 1% Triton X-100 (PBT) (in a 1.5 mL Eppendorf tube) and rotated at room temperature for 30 minutes. After washing twice for 5 minutes each in PBT, blocking solution of PBT containing 10% bovine serum albumin (10% BSA) was applied to samples and rotated at room temperature for 1 hour. Samples were washed in PBT containing 1% bovine serum albumin (1% BSA) for 5 minutes. Primary Rabbit anti-ATP-synthase antibody was then applied at a dilution of 1:400. Samples were incubated in primary antibody overnight at 4 degrees on nutator. After that, samples were washed in PBT with 1% BSA solution three times for 10 minutes each. Samples were incubated with appropriate secondary antibody at room temperature on rotator for two hours. The following secondary antibodies were used: Goat Anti-Rabbit Alexa 568, Goat Anti-Rabbit Alexa 488 and Alexa 633 (from Molecular Probes) at a dilution of 1:400. Next, samples were washed twice in PBT buffer for 5 minutes and then subjected to chromatin staining using Draq5 (Biostatus) at a dilution 1∶500 in PBT buffer for 1 hour at room temperature on nutator or OliGreen (Invitrogen) at a dilution of 1∶10,000 in PBT buffer solution for 10 minutes at room temperature. Samples stained with OliGreen were then washed twice for 5 minutes in PBT buffer solution and fixed to microscope slide. Images were obtained using the Leica (DM-IRB) Confocal System.

### Apoptosis detection

We used the ApopTag® Fluorescein In Situ Apoptosis Detection Kit (Millipore # S7110) to detect the occurrence of cell death in first instar larvae brains. Tissues from wild type or larvae expressing *Tim17b^RNAi^* were dissected in Grace's, fixed as described above, and washed 10 min in PBT. Tissues were processed according to the manufacturer's recommendations, then rinsed with PBS, and immunostained as described above.

**Imaging of Live** ***Drosophila*** **larval tissues**, as described in [32].

### Fluorescence Recovery After Photobleaching (FRAP) assay

FRAP experiments on live *Drosophila* tissues were performed as described in [46]. To conduct these experiments, we used a Leica TCS SP2 confocal microscope with capacity for FRAP. To avoid oxidative stress and other damage sometimes caused by lasers, we used only the minimal level of laser power. This step extended the “bleaching” phase, but it did not affect our results. To collect FRAP data, we employed the “FlyMode” program, which allows data collection, even during the bleaching phase. Recordings were performed via a 63×1.4 NA oil immersion objective. Previously, we found that all the fluorescent epitopes we tested (ECFP, EYFP (Venus), EGFP, and DsRed) were appropriate for FRAP assays [47], as well as for regular confocal analysis. We did not detect epitope-specific biases in the function, expression dynamics or localization of any fused moiety. We used transgenic fly stocks that expressed appropriate fluorescent epitope-tagged protein. Tissues were dissected in Grace's Media, and dynamic movement of fluorescent proteins was analyzed for 20–30 minutes following dissection.

## Supporting Information

Figure S1 **Evolutionary conservation for Tim17b protein in eukaryotes.** **A.** Comparison of amino acid sequences of Tim17 proteins from yeast (TIM17_YEAST), *C. savinguvi* (ENSCSAVT00000007450_CIOSA), *D. persimilis* (dper_GLEANR_15855_ caf1_DROPE), *C. briggsae* (CBG01742_CAEBR), Rat (Timm17b_predicted_RAT), Mouse (Timm17b_MOUSE), Bovine (TI17B_BOVIN), *Canis familiaris* (TIMM17B_CANFA), Horse (TIMM17B_HORSE), Human (TIMM17B_HUMAN), Chicken (NP_001026197_CHICK) and *D. melanogaster* (gi|32330112|gb|ADX35898.1). Evolutionary conservation and consensus sequence are shown below. **B.** Alignment of three Tim17b homologues from *Drosophila melanogaster* genome: Tim17b (gi|323301112|gb|ADX35898.1| MIP28909p); Tim17b1 (gi|24644195|ref|NP_649526.2|); and Tim17b2 (gi|24584449|ref|NP_524746.2|). **C.** Expression profiles of three Tim17b homologous proteins during *Drosophila* development. RT-PCR using gene-specific primers demonstrates that Tim17b1 and Tim17b2 express predominantly in adult males, while Tim17b is ubiquitous in each tested developmental stage. Primers specific to Tubulin mRNA were used as a loading control.
(TIF)
