# Supplementary figures and images for: Generating a Knockdown Transgene against Drosophila Heterochromatic Tim17b Gene Encoding Mitochondrial Translocase Subunit
Source: PLoS One. 2011 Oct 6;6(10):e25945. doi: 10.1371/journal.pone.0025945 (PMC3188573; doi:10.1371/journal.pone.0025945)

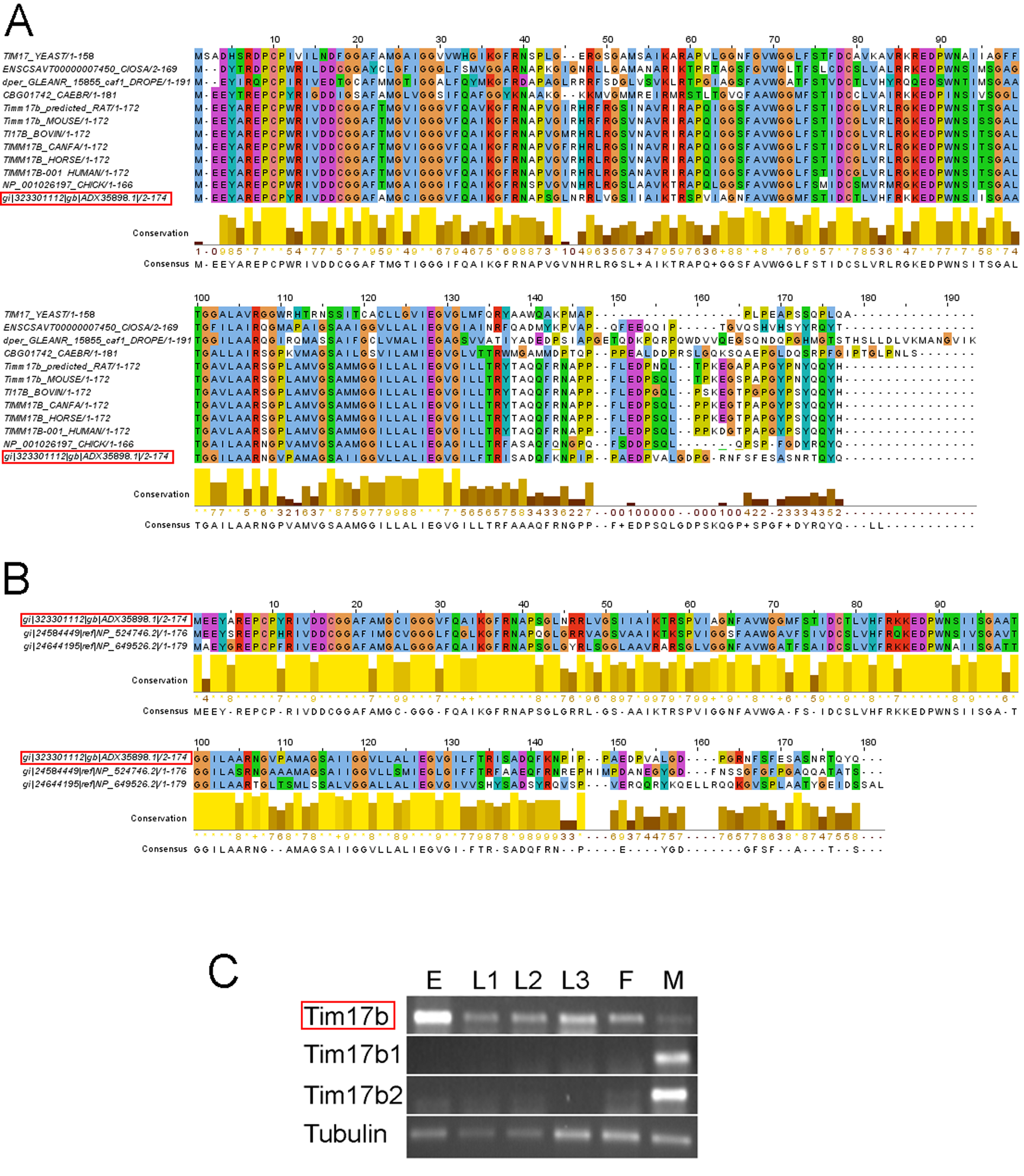

Supplement: Figure S1 — Evolutionary conservation for Tim17b protein in eukaryotes. A. Comparison of amino acid sequences of Tim17 proteins from yeast (TIM17_YEAST), C. savinguvi (ENSCSAVT00000007450_CIOSA), D. persimilis (dper_GLEANR_15855_ caf1_DROPE), C. briggsae (CBG01742_CAEBR), Rat (Timm17b_predicted_RAT), Mouse (Timm17b_MOUSE), Bovine (TI17B_BOVIN), Canis familiaris (TIMM17B_CANFA), Horse (TIMM17B_HORSE), Human (TIMM17B_HUMAN), Chicken (NP_001026197_CHICK) and D. melanogaster (gi|32330112|gb|ADX35898.1). Evolutionary conservation and consensus sequence are shown below. B. Alignment of three Tim17b homologues from Drosophila melanogaster genome: Tim17b (gi|323301112|gb|ADX35898.1| MIP28909p); Tim17b1 (gi|24644195|ref|NP_649526.2|); and Tim17b2 (gi|24584449|ref|NP_524746.2|). C. Expression profiles of three Tim17b homologous proteins during Drosophila development. RT-PCR using gene-specific primers demonstrates that Tim17b1 and Tim17b2 express predominantly in adult males, while Tim17b is ubiquitous in each tested developmental stage. Primers specific to Tubulin mRNA were used as a loading control. (TIF) [file pone.0025945.s001.tif]
